# Ebselen Interferes with Alzheimer’s Disease by Regulating Mitochondrial Function

**DOI:** 10.3390/antiox11071350

**Published:** 2022-07-11

**Authors:** Xuexia Li, Qingqing Shi, Hao Xu, Yufang Xiong, Chao Wang, Linfeng Le, Junliang Lian, Guoli Wu, Feiyuan Peng, Qiong Liu, Xiubo Du

**Affiliations:** 1 Guangdong Provincial Key Laboratory for Plant Epigenetics, College of Life Sciences and Oceanography, Shenzhen University, Shenzhen 518055, China; xxli@szu.edu.cn (X.L.); 2019302013@email.szu.edu.cn (H.X.); 2019305009@email.szu.edu.cn (Y.X.); 2019301033@email.szu.edu.cn (L.L.); 2018304045@email.szu.edu.cn (J.L.); 2020302016@email.szu.edu.cn (G.W.); 2021300031@email.szu.edu.cn (F.P.); liuqiong@szu.edu.cn (Q.L.); 2 Key Laboratory of Optoelectronic Devices and Systems of Ministry of Education and Guangdong Province, College of Physics and Optoelectronic Engineering, Shenzhen University, Shenzhen 518060, China; 3 Shenzhen-Hong Kong Institute of Brain Science—Shenzhen Fundamental Research Institutions, Shenzhen 518055, China; 4 Shenzhen Bay Laboratory, Shenzhen 518055, China; 5 Department of Psychiatry, Xijing Hospital, Air Force Medical University, Xi’an 710032, China; pengzw@fmmu.edu.cn; 6 Shenzhen Center for Disease Control and Prevention, Shenzhen 518055, China; lihua@wjw.sz.gov.cn

**Keywords:** Alzheimer’s disease, Ebselen, synapse, Aβ, mitochondria

## Abstract

(1) Background: With unknown causes and no effective treatment available, Alzheimer’s disease (AD) places enormous pressure on families and society. Our previous study had shown that Ebselen at a high concentration (10.94 μM) improved the cognition of triple-transgenic AD (3×Tg-AD) mice and alleviated the related pathological indicators but showed toxicity to the mice. Here, we dedicated to study the therapeutic effect and molecular mechanism of Ebselen at a much lower concentration on 3×Tg-AD mice. (2) Methods: Various behavioral experiments were applied to detect the behavioral ability of mice. Western blot, thioflavin T staining and a transmission electron microscope were used to evaluate the pathology of AD mice. The mitochondrial membrane potential and respiration were assessed with the corresponding assay kit. (3) Results: Ebselen remarkably increased cognitive ability of AD mice, eliminated β-Amyloid (Aβ) oligomers and recovered the synaptic damage in AD mice brain. In addition, the destroyed mitochondrial morphologies and function were repaired by Ebselen through ameliorating mitochondrial energy metabolism, mitochondrial biogenesis and mitochondrial fusion/fission balance in N2a-SW cells and brain tissues of AD mice. (4) Conclusions: This research indicated that Ebselen might exert its therapeutic effect via protecting mitochondria in AD.

## 1. Introduction

Since the 20th century, the aging problem has become increasingly serious, and the main factors posing serious challenges to families and society are age-related neurodegenerative diseases, especially Alzheimer’s disease (AD). Patients with AD are characterized by progressive deterioration of learning and memory and various neurological and behavioral disorders such as aphasia and apraxia [1]. As an increasingly prevalent disease, AD has been a big healthcare, financial and caregiver burden [2]. However, the etiology and pathogenesis of AD are not fully clear, and there is no effective drug either.

As an essential trace element, selenium (Se) has a broad range of pharmacological activities, including antioxidation, anti-aging, anti-cancer, regulating protein synthesis, enhancing human immunity, etc. [3,4]. Based on these biological functions of selenium, we and others have shown that selenium, whether as a selenium compound or a selenoprotein, has a close relationship with AD. Previously, we reported that se-methylselenocysteine (SMC) ameliorated AD-related pathologies by reducing oxidation, regulating metal homeostasis and inhibiting the extracellular signal-regulated kinase (ERK) pathway [5]. Another organic selenium, selenomethionine (Se-Met), was found to attenuate the amyloid and hyperphosphorylated tau levels, modulate autophagy, reduce glial activation, attenuate neuroinflammation, relieve nerve cell death and reverse synaptic deficit in AD model cells and mice [6,7,8,9]. In addition, severe selenium deficiency was detected in both AD patients and AD model mice [10,11,12]. These studies suggest that selenium, as an essential micronutrient, has a potential role in the treatment of AD.

Ebselen, developed as an organic selenium-containing compound, shows lower toxicity than inorganic selenium does. With anti-inflammatory and antioxidant properties, Ebselen can catalyze some basic reactions in the body to protect cellular components from oxidative stress. Ebselen is also able to bind to the cysteine of several proteins and has been considered to be a safe lithium analog and to protect against several microorganisms [13,14]. Because of the effects, Ebselen has potential in the prevention and treatment of some diseases such as arthritis, atherosclerosis, stroke, neurodegenerative disease, cancer and COVID-19 [15,16]. In addition, Ebselen is able to cross the blood–brain barrier (BBB) and keep pharmacological activity in the brain [13,17], making it an interesting compound in AD drug development. It has been reported that Ebselen reduces hippocampal oxidation, ameliorates apoptosis, ameliorates cell proliferation and improves memory of AD mice [12,18,19].

Our previous research found that two-month-old 3×Tg-AD mice treated with 3 μg/mL (10.94 μM) Ebselen for six months performed significantly better than untreated AD controls did. β-Amyloid (Aβ), tau and synaptic pathology in AD mice were markedly reversed, implying that Ebselen had a better preventive effect on AD [20]. Since Ebselen has been shown to have significant cytotoxicity, it is necessary to conduct appropriate studies to design the optimal dose of Ebselen for clinical trials [15,21]. To explore the therapeutic effect and mechanism of Ebselen on AD pathologies, 3×Tg-AD mice were administered much lower concentrations of Ebselen (0.5, 1 and 2 μM) at the age of four months when the cognitive impairment begins to appear [22]. After five months of treatment with Ebselen, 3×Tg-AD mice were tested by a series of behavioral experiments and sacrificed for mechanism exploration. The results showed that in the absence of toxicity, low concentrations of Ebselen significantly intervened with the pathology of AD mice by alleviating mitochondrial damage.

## 2. Materials and Methods

3×Tg-AD mice harboring TauP301L, PS1M146 V and APPswe were purchased from Jackson laboratory (Bar Harbor, ME, USA). The animal experiments were conducted according to the guidelines of the Chinese Council on Animal Care and approved by the Animal Ethical and Welfare Committee of Shenzhen University (Permit Number: AEWC-20140615-002). A detailed description of materials and methods is provided in Supplementary Materials.

## 3. Results

### 3.1. Ebselen Promoted Glutathione Peroxidase Activities of N2a-SW Cells

Ebselen is an important mimic of glutathione peroxidases (GPx), the selenium-dependent hydroperoxidase-reducing enzymes that contribute in reducing H_2_O_2_ and fatty acid hydroperoxides. As shown in Figure S1A, GPx activity of N2a-SW cells was lower than that of N2a cells, although with no significant difference between them. After Ebselen treatment, GPx activity of N2a-SW cells increased significantly, especially at the moderate concentration (2.5 μM). Furthermore, the expression levels of GPX1 and GPX4 in N2a-SW cells were significantly lower than those in N2a cells (Figure S1B). There was no significant difference in the level of GPX1 protein between Ebselen-treated and nontreated N2a-SW cells. Meanwhile, 2.5 and 5 μM Ebselen notably increased GPX4 level of N2a-SW cells. These results indicated that Ebselen could increase GPx activity and level in AD model cells.

### 3.2. The Effect of Ebselen on Serum Biochemical Indicators

**Figure 1:**
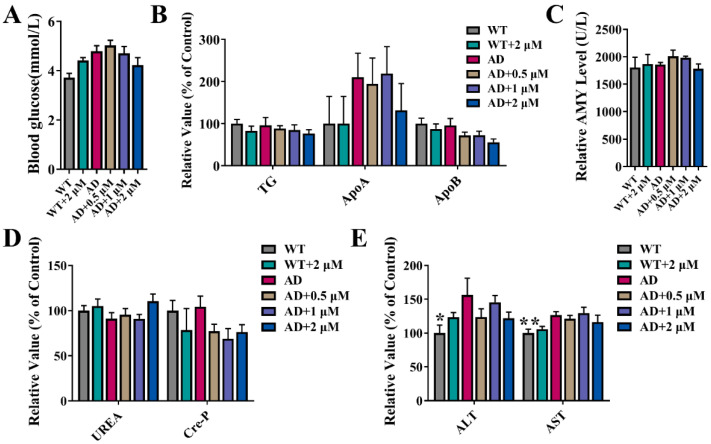
Effect of Ebselen on biochemical indicators of 9-month-old AD mice: (**A**) The level of blood glucose; (**B**) relative levels of blood-lipid-related biochemical indicators, including triglyceride (TG), apolipoprotein A (ApoA) and apolipoprotein B (ApoB); (**C**) the level of α-amylase (AMY); (**D**) relative levels of kidney-related biochemical indicators, including urea (UREA) as well as creatinine (Cre-P). (**E**) Relative levels of liver-related biochemical indicators, including glutamine transferase (ALT) and aspartate transferase (AST). (* *p* < 0.05, ** *p* < 0.01 vs. AD group; *n* = 12).

In order to explore the toxicity of Ebselen to mice, several biochemical indicators in the serum of the mice were analyzed. The results showed that there were no significant differences in the levels of blood glucose, blood-lipid-related biochemical indicators, including triglyceride (TG), apolipoprotein A (ApoA) and apolipoprotein B (ApoB), α-amylase (AMY), the main biochemical indicator of pancreas injury, and urea (UREA) and creatinine (Cre-P), the main biochemical indicators reflecting the metabolic capacity of the kidney (Figure 1A–D). Meanwhile, the levels of glutamine transferase (ALT) and aspartate transferase (AST), two biochemical indicators reflecting liver function, were notably higher in 3×Tg-AD mice compared with those in WT mice. Ebselen of 2 μM decreased the levels of ALT and AST to a certain extent, although without a significant difference (Figure 1E). These results indicated that Ebselen of 0.5–2 μM had no significant effect on blood glucose and lipid levels and showed no toxicity to the pancreas, kidney and liver of AD mice.

### 3.3. Ebselen Rescued Behavioral Deficits of 3×Tg-AD Mice

#### 3.3.1. Ebselen Rescued Memory Deficits of 3×Tg-AD Mice

**Figure 2:**
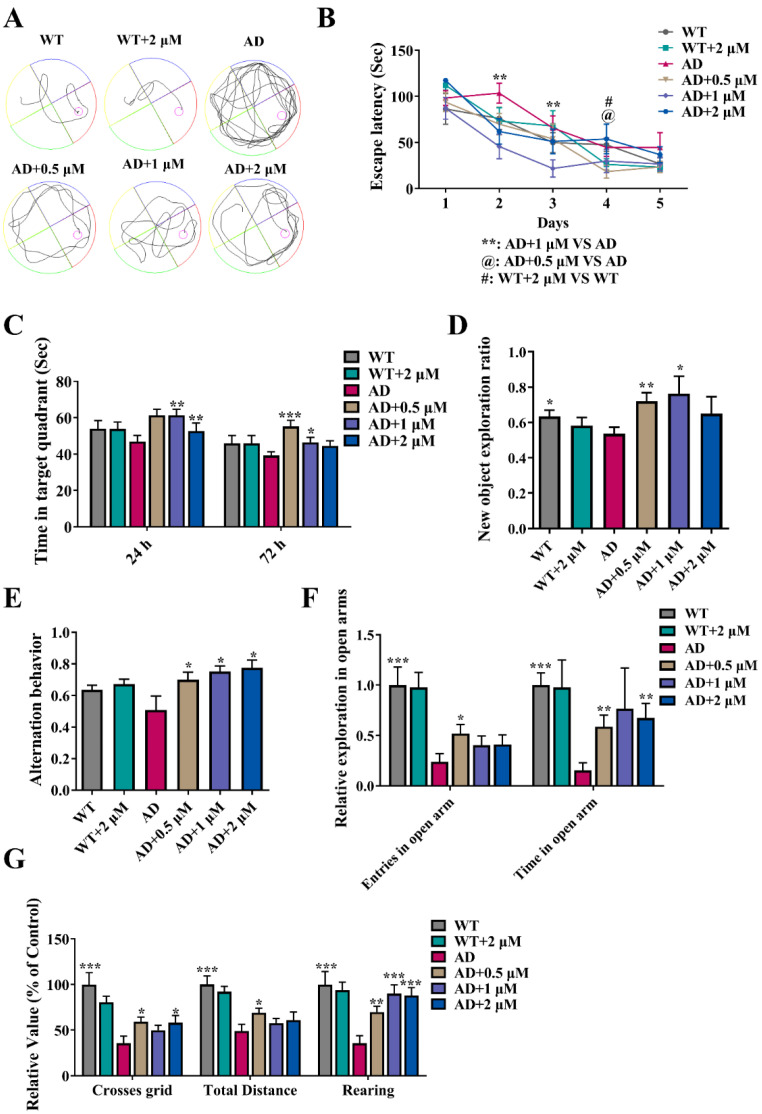
Ebselen administration improved cognitive deficits of 9-month-old AD mice: (**A**–**C**) Morris water maze involving the representative swimming trajectory for mice on Day 5 in a learning trial (**A**), the mean latency of mice in each trial day (**B**) and time spent in the effective quadrant in probe trials (**C**); (**D**) new object exploration ratio tested by Novel Object Recognition Test. (**E**) Y Maze Test to determine alternation behavior; (**F**) Elevated Plus Maze Test including the entries in the open arms, and the total time stayed in the open arms; (**G**) Open Field Test including the numbers of crossed grids, total distance and rearing frequencies. (* *p* < 0.05, ** *p* < 0.01, *** *p* < 0.001 vs. AD group; *n* = 12).

To examine the therapeutic efficacy of Ebselen, a Morris water maze was performed to detect the cognitive ability of mice. As illustrated in Figure 2A, the representative swimming trajectory on Day 5 in the spatial learning trial indicated that AD mice showed a longer escape latency compared with that of WT mice. However, the prolonged latency was greatly shortened by Ebselen treatment. As seen in Figure 2B, the escape latencies of all groups were gradually shortened over five consecutive training days. From Day 2 to Day 4, 1 μM-Ebselen-treated AD mice performed obviously better than AD mice did. In addition, 24 and 72 h after the spatial learning trial, mice treated with 0.5 or 1 μM Ebselen stayed in the quadrant where the target platform had been located significantly longer than AD mice did (Figure 2C). The results indicated that the mice treated with Ebselen reserved memory ability better than that of AD mice. Next, the Novel Object Recognition Test was used to further assess the memory and exploratory ability of AD mice. As shown in Figure 2D, the new object exploration ratio of AD mice was remarkably lower than that of WT mice. AD mice and 2 μM-Ebselen-treated AD mice did not differ significantly. Meanwhile, 0.5 and 1 μM Ebselen remarkably increased the new object exploration ratio of AD mice. However, there was no significant difference in the total number of explorations of both objects between all groups of mice (Figure S2A). Spontaneous alteration rate in the Y Maze Test was aimed to evaluate the spatial working memory, and a higher value means better memory. The results showed that the spontaneous alternations rate of Ebselen-treated mice was remarkably higher than that of AD mice (Figure 2E), although there was no difference in the total arm entries between Ebselen-treated mice and AD mice (Figure S2B).

#### 3.3.2. Ebselen Significantly Improved Locomotor and Exploring Ability and Alleviated the Anxiety of AD Mice

Next, the Elevated Plus Maze Test as well as the Open Field Test were used to test the exploration ability and anxiolytic activity of AD mice after five months of Ebselen treatment. The Elevated Plus Maze Test demonstrated that the entries and the total time spent in the open arms of AD mice were notably decreased compared with those of WT mice, which were both increased after 0.5 μM Ebselen treatment (Figure 2F). Furthermore, 2 μM Ebselen-treated mice also showed an obvious increase in the total time spent in the open arms (Figure 2F). Furthermore, compared with those of WT mice, AD mice exhibited significant reductions in the numbers of crossed grids, total distance and frequencies of rearing (Figure 2G) in the Open Field Test. Meanwhile, 0.5 and 2 μM Ebselen markedly increased the number of crossed grids of AD mice. Ebselen of 0.5 μM also notably increased the total exploration distance of AD mice. All Ebselen-treated mice reared more frequently than AD mice did (Figure 2G). The results suggested that Ebselen significantly increased the locomotor and exploring ability of AD mice.

### 3.4. Ebselen Restored the Reduction of Synaptic Protein Levels and Repaired Synaptic Morphologies of AD Mice

**Figure 3:**
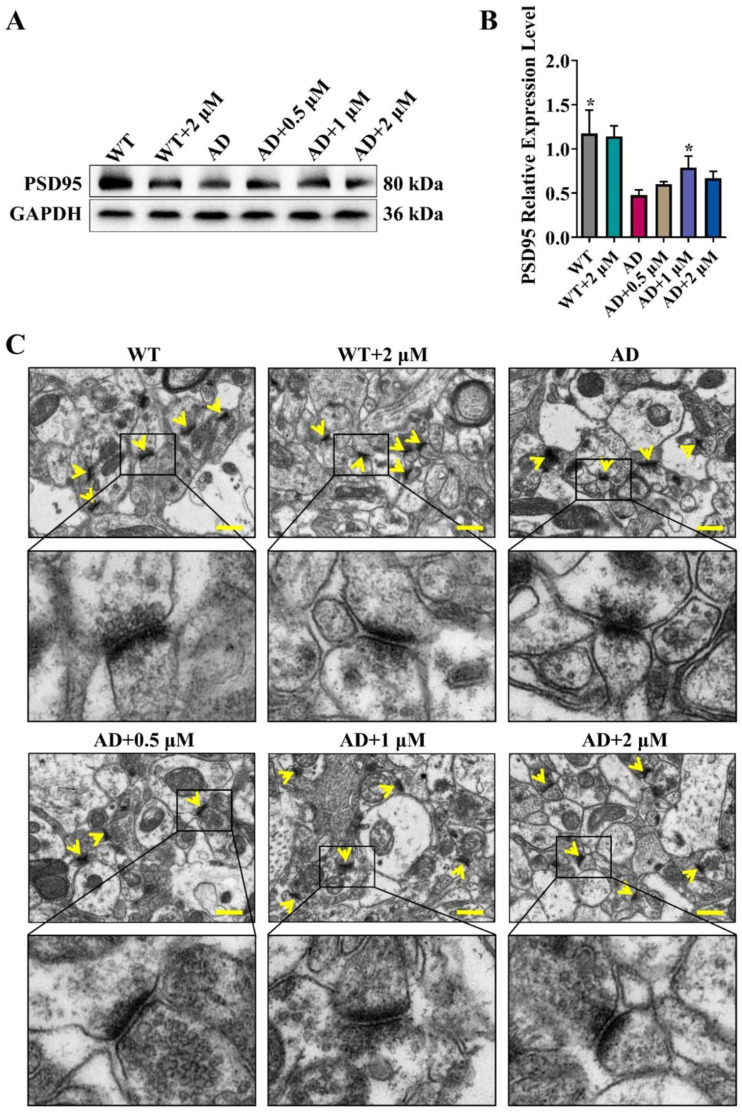
Ebselen increased synaptic protein levels and repaired synaptic morphologies in AD mice brains: (**A**,**B**) Representative Western blot analysis of PSD95. The quantitative results were normalized by GAPDH (* *p* < 0.05 vs. AD group; *n* = 3). (**C**) Representative transmission electron microscopic images of synapses (yellow arrows) in the cerebral cortex of mice. (Scale bar: 5.0 μm; *n* = 3).

Synaptic loss and impairment are the main pathological bases of cognitive impairment in AD. Our previous study had shown that Ebselen treatment preserved the expressions of postsynaptic density protein 95 (PSD95) as well as Synaptophysin (Syna) in N2A-SW cells [20]. Consistently with the results of N2a-SW AD cell models, 1 μM Ebselen treatment significantly increased cortical PSD95 levels in AD mice (Figure 3A,B). Meanwhile, we directly assessed the synaptic morphologies in AD mice brains by a transmission electron microscope. As seen in Figure 3C, the synaptic structure of AD mice had edema, the synaptic vesicles were damaged seriously and the postsynaptic membrane and the synaptic space were blurred and melted. After Ebselen treatment, the synapses of AD mice were significantly repaired, i.e., the synapse structure became intact and the presynaptic membrane, postsynaptic membrane and the synaptic space were distinguishable. 

### 3.5. Ebselen Inhibited Aβ Pathologies in AD Mice Brains

**Figure 4:**
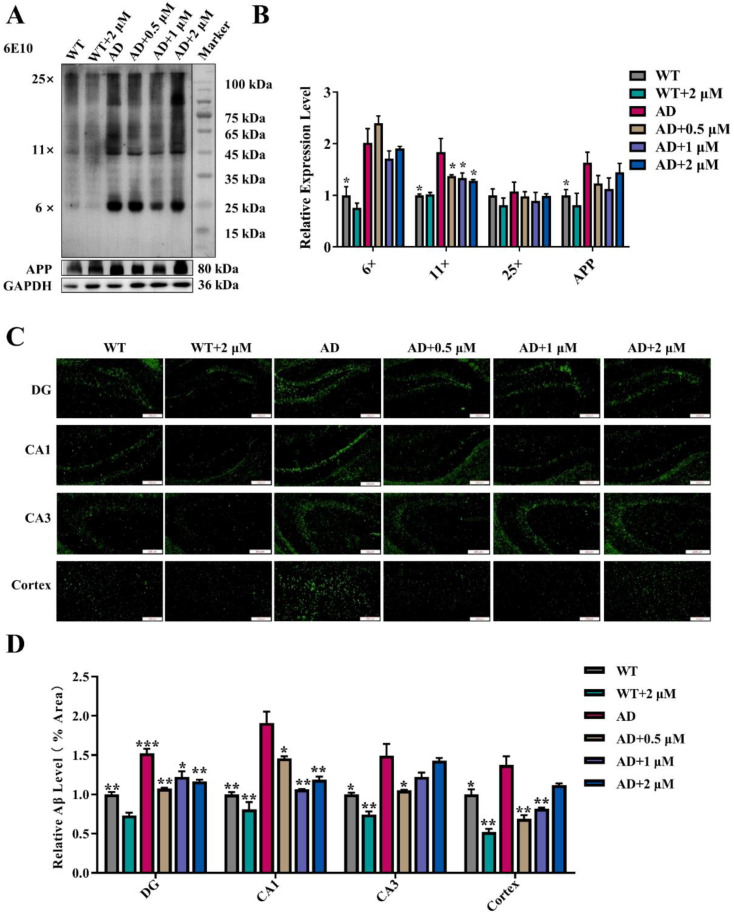
Ebselen inhibited Aβ pathologies in AD mice brains: (**A**,**B**) Representative Western blot analysis of various Aβ oligomers (6×, 11×, and 25×) and APP. The quantitative results were normalized by GAPDH. (**C**) Thioflavin T staining to detect the level and distribution of amyloid plaques in brain tissues (Scale bar: 200 μm); (**D**) quantitative analysis of thioflavin T staining. (* *p* < 0.05, ** *p* < 0.01, *** *p* < 0.001 vs. AD group; *n* = 3).

Previously, we found that 3 μg/mL (10.94 μM) Ebselen reduced the level of Aβ oligomers by downregulating the expression of both amyloid precursor protein (APP) and β-Site amyloid precursor protein cleaving enzyme 1 (BACE1) in AD mice brains [20]. Here, we found that when the concentration was reduced to 0.5–2 μM, Ebselen could also reduce the levels of Aβ oligomers (8×, 11× and 25×) and APP in AD mice brains (Figure 4A,B). In agreement with this, thioflavin T staining showed that amyloid plaques in CAI, CA3, DG and the cortex of AD mice were much more than those in WT mice, and Ebselen reduced Aβ deposition in CAI, DG and the cortex of AD mice (Figure 4C,D).

### 3.6. Ebselen Alleviated the Structural and Functional Damage of Mitochondria in AD Models

#### 3.6.1. Ebselen Rescued Mitochondrial Health

**Figure 5:**
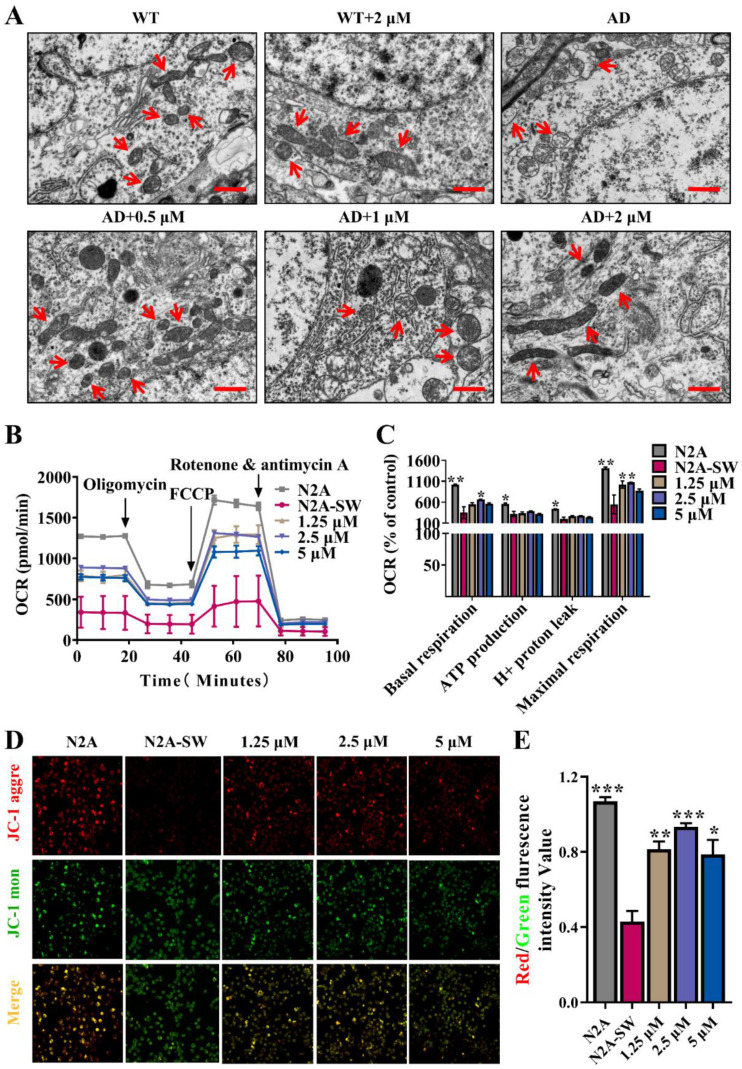
Effect of Ebselen on mitochondrial state: (**A**) Transmission electron microscopic images of mitochondria (red arrows) in the cerebral cortex of mice (scale bar: 10 μm; *n* = 3). (**B**,**C**) Mitochondrial respiration of N2a-SW cells, including the real-time change of OCR levels in cells, (**B**) and key parameters of mitochondrial function obtained from the OCR curve involving basal respiration, ATP production, H^+^ protein leak and maximal respiration (**C**). (**D**,**E**) Cellular ΔΨm measured by a JC-1 fluorescent probe, including representative images of cells stained with JC-1 (**D**), and red/green fluorescence intensity (**E**). (* *p* < 0.05, ** *p* < 0.01, *** *p* < 0.001 vs. AD group; *n* = 4).

Mitochondrial damage is an early pathological event in AD. Here, we directly observed the ultrastructure of mitochondria by transmission electron microscopy. As illustrated in Figure 5A, in comparison with those of WT mice, the mitochondria of AD mice were damaged seriously, were fewer, inhomogeneous, swollen and vacuolated; the electron density of the mitochondrial matrix was decreased, and the mitochondrial crest dilated and appeared vague. In addition, the mitochondria in AD mice were irregular with low electron density and an incomplete membrane. Meanwhile, after the treatment with Ebselen, the defects of mitochondrial structure were alleviated, especially at low doses (1.25 and 0.5 μM). Consistently with the result shown in an AD mouse model, Ebselen restored the morphological damage of mitochondria at the cellular level (Figure S3).

The functional state of mitochondria is closely related to its morphology. To test the effects of Ebselen on mitochondrial function, the mitochondrial respiration of N2a-SW cells was assessed via the Agilent Seahorse XF Cell Mito Stress Test Kit. The oxygen consumption rate (OCR) with sequential injection of oligomycin (the ATP synthase inhibitor), FCCP (the mitochondrial uncoupler) and rotenone + antimycin A (the mitochondrial ETC inhibitor) are depicted in Figure 5B. Basal respiration, ATP production, H+ proton leak and maximal respiration were analyzed based on the OCR curves (Figure 5C). The results demonstrated that all these indicators of N2a-SW cells decreased compared with those in N2a cells, reflecting the perturbations in the mitochondrial respiration of N2a-SW cells. Interestingly, we found that 2.5 μM Ebselen significantly improved the basal respiration. Furthermore, 1.5 μM as well as 2.5 μM Ebselen notably enhanced the maximal respiration of N2a-SW cells. 

ΔΨm, whose depolarization indicated mitochondrial dysfunction, was then measured by a JC-1 fluorescent probe. Under physiological conditions, JC-1 was in the aggregated form, localized in the matrix of the mitochondria and emitted red fluorescence, indicating high polarized mitochondria. Meanwhile, in the pathological condition, JC-1 was in the monomeric form, localized in the cytoplasm and emitted green fluorescence, indicating low polarized mitochondria. Therefore, ΔΨm could be determined by the ratio of red-to-green fluorescence intensities. As showcased in Figure 5D,E, the lower ΔΨm in N2a-SW cells compared with that in N2a cells was notably increased by Ebselen.

#### 3.6.2. Ebselen Repaired the Mitochondria by Improving Mitochondrial Energy Metabolism, Enhancing Mitochondrial Biogenesis and Balancing Mitochondrial Fission/Fusion

**Figure 6:**
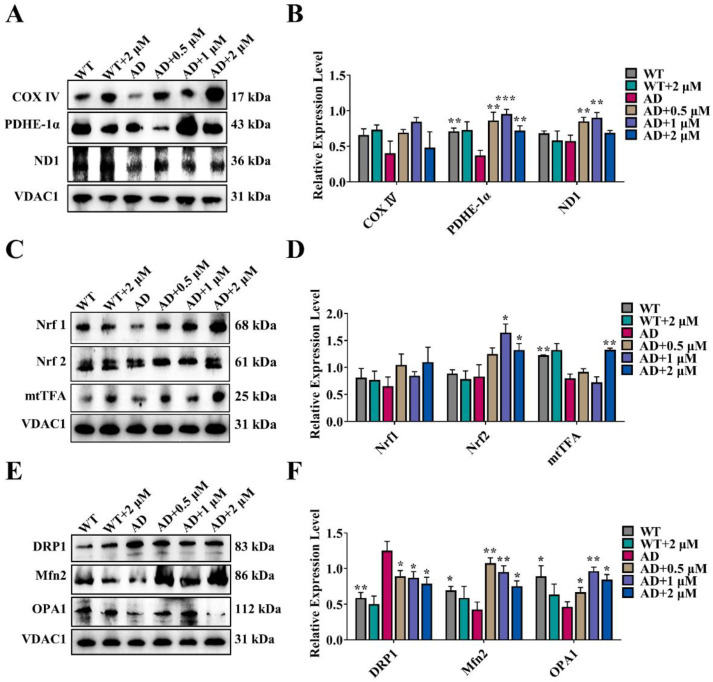
Ebselen improved mitochondrial energy metabolism, enhanced mitochondrial biogenesis and balanced mitochondrial fission/fusion in the cerebral cortex of mice: (**A**,**B**) Representative Western blot analysis of mitochondrial energy-metabolism-related proteins, including COX IV, PDHE-1α and ND1. (**C**,**D**) Representative Western blot analysis of mitochondrial biogenesis-related proteins, including Nrf1, Nrf2 and mtTFA. (**E**,**F**) Representative Western blot analysis of mitochondrial dynamics-related proteins, including the fission protein DRP1 and the fusion proteins Mfn2 and OPA1. The quantitative results were normalized to GAPDH. (* *p* < 0.05, ** *p* < 0.01, *** *p* < 0.001 vs. AD group; *n* = 4).

To investigate the effect of Ebselen on mitochondrial energy metabolism, the expression levels of cytochrome c oxidase IV (COX IV), pyruvate dehydrogenase-E1α (PDHE-1α) and NADH dehydrogenase 1 (ND1) were determined both in vitro and in vivo. As showcased in Figure 6A,B and Figure S4A,B, the levels of COX IV, PDHE-1α and ND1 in AD groups were significantly lower compared with those in the control group. After treatment with Ebselen, their levels were upregulated. 

Inhibition of mitochondrial biogenesis in AD can lead to a series of disorders of mitochondrial function. As shown in Figure 6C,D and Figure S4C,D, expression levels of factors regulating mitochondrial biogenesis, including nuclear respiratory factor 1 (Nrf1), nuclear respiratory factor 2 (Nrf2) and mitochondrial transcription factor A (mtTFA) were severely decreased in both AD cortical tissue and N2a-SW cells. This situation was reversed by Ebselen to a certain extent, suggesting that Ebselen enhanced the process of Mitochondrial biogenesis.

Mitochondria are highly dynamic organelles via a process of fusion and fission dynamics. Abnormal mitochondrial dynamics are particularly relevant to the occurrence and development of AD [23]. Thus, the expression levels of mitochondrial fusion proteins involving mitofusin2 (Mfn2) and optic atrophy 1 protein (OPA1) and mitochondrial fission protein dynamin-related protein 1 (DRP1) were tested both at cellular and animal levels. As shown in Figure 6E,F and Figure S3E,F, the level of DRP1 was markedly increased, and mitochondrial-fusion-related proteins (Mfn2 and OPA1) were decreased in AD groups. After Ebselen treatment, mitochondrial fission reduced and mitochondrial fusion increased both in vitro and in vivo.

## 4. Discussion

Selenium has been studied for decades and seems to be associated with most molecular mechanisms involved in neurodegenerative diseases, especially AD. Ebselen (also known as PZ51) was designed and synthesized to mimic Glutathione peroxidase (GPx) and proved to have strong antioxidant activity [24]. Previously, we reported that a long-term treatment (six months) with high-dose Ebselen (10.94 μM) to two-month-old 3×Tg-AD mice significantly prevented the progression of AD-related pathology, implying the good preventive effect of Ebselen on AD [20]. However, Ebselen can be toxic in vitro and in vivo when administered at high doses [15]. It has been demonstrated that high concentrations of Ebselen decreased the viabilities of cells, increased the damage index of DNA, interfered with Ca^2+^ homeostasis in cells, induced depolarization of mitochondrial membrane potential, increased mitochondrial membrane permeability and disrupted the mitochondrial network [25,26,27]. Furthermore, 75 μM Ebselen reduces ALT activity by about 25% in rodents, and 60 μM Ebselen increases the serum urea level by about 40%, indicating that a high dose of Ebselen induces liver and kidney damage in rodents [28]. In our previous study, we observed that 10.94 μM Ebselen caused hairlessness and hepatosplenomegaly in mice, although it improved cognitive impairment in AD mice. In order to clarify the therapeutic effect of Ebselen at a low dose on AD and the underlying mechanism, 3×Tg-AD mice at four months were administered Ebselen (0.5–2 μM) when cognitive impairment began to appear [22]. After five months of treatment with Ebselen, AD mice were tested via various behavioral experiments and sacrificed for following mechanism exploration. As shown in Figure 1B–D, Ebselen at the above concentrations did not affect the important serum indicators of pancreas, kidney and liver injury, indicating that Ebselen at these concentrations was relatively safe to mice.

As a GPx mimic, the influence of Ebselen on GPx activities of AD model cells was evaluated. GPx-1 and GPx-4 are the two main types of GPx with antioxidant capacity. GPx-1 can reduce H_2_O_2_ to H_2_O and O_2_ and plays a key role in keeping mitochondria healthy and a normal thiol redox balance [29]. GPx-4 is primarily known for lipid peroxidation and ferroptosis and also has an inhibitory effect on neurodegeneration [30]. In this study, the total GPx activity and the levels of GPx-1 and GPx-4 in N2a-sw cells were found to be much lower than those in N2a cells and significantly reversed by Ebselen, especially at the moderate concentration (2.5 μM) (Figure S1). Next, the therapeutic effect of Ebselen on the cognitive deficit of AD mice was detected by various behavioral experiments. It was revealed that 0.5 and 1 μM Ebselen was effective in rescuing spatial learning and memory deficits of AD mice (Figure 2A–E). In addition, low dosage (0.5 μM) of Ebselen significantly improved the spontaneous activity and relieved the anxiety of AD mice (Figure 2F,G). Previously, we found that high dosage of Ebselen (10.94 μM) improved the cognition of 3×Tg-AD mice [20], therefore, it could be concluded that even though the concentration of Ebselen was reduced 20 times (from 10.94 to 0.5 μM), it still showed a therapeutic effect on the cognitive deficit of AD mice. These observations indicated that Ebselen effectively improved the exploratory and spontaneous activity of AD mice and provided a dose basis for the application of selenium compounds in clinical trials. 

Aβ pathology is one of the key histopathological hallmarks of AD and damages neurons and synapses and consequently impairs the behavioral and cognitive performance of AD mice. As illustrated in Figure 4, Ebselen, especially at the moderate concentration, not only reduced Aβ oligomers levels, but also decreased Aβ deposition in AD mice brains. Consequently, the synaptic structure was reversed, and the protein levels of PSD95, a key synaptic protein, were also increased in AD mice brains upon the treatment with Ebselen (Figure 3).

The mitochondrion is an organelle with a double membrane and responsible for the supply of the majority of energy for cells, especially for neurons with exceptionally high and continuous energy requirements. One of the most obvious characteristics of the brain is its huge energy demand, thus, mitochondrial dysfunction will inevitably affect brain function. With the continuous research on AD pathology, mitochondrial disorder has been regarded as an early typical characteristic of AD [31]. Here, we found that Ebselen at lower doses significantly improved the morphological damage, promoted respiration and increased ΔΨm of mitochondria (Figure 5).

COX IV is a key subunit of cytochrome C oxidase, encoded by the nucleus and related to mitochondrial respiration. Decreased levels of COX IV in an organism will affect mitochondrial respiration, resulting in reduced ATP production [32]. PDHE-1α is one of the core subunits of pyruvate dehydrogenase and participates in the biochemical reactions such as the TCA cycle and oxidative phosphorylation. When PDHE1 is lacking, the aerobic oxidation in the organism is inhibited, resulting in an increase of lactic acid and a decrease of ATP level [33]. In addition, NADH dehydrogenase 1 (ND1) is also related to mitochondrial respiration, and its decrease also causes mitochondrial dysfunction [34]. In this study, we found that the levels of COX IV, PDHE-1α and ND1 in AD model cells/mice were decreased and notably reversed by Ebselen (Figure 6A,B). The result suggested that Ebselen could maintain the energy supply of mitochondria to neurons of AD mice, which might then slow down further development of AD. 

Transcription and signaling cascades of mitochondrial biogenesis is important in neuronal development and neurodegenerative diseases [35]. After binding to the mtTFA promoter, NRF-1 and NRF-2 regulatory factors activate mtDNA transcription of mtTFA and indirectly regulate the expression of the three subunits of the mitochondrial COX complex, thereby regulating mitochondrial energy metabolism [36]. In the present research, after Ebselen treatment, the levels of NRF-1 and NRF-2 in AD models were obviously increased, thereby increasing the level of mtTFA (Figure 6C,D). The results indicated that Ebselen could trigger mitochondrial biogenesis, thus maintaining mitochondrial function.

Mitochondrial dynamics is a general term for the processes of mitochondrial fission/fusion, occurrence, cleavage and transportation, which controls mitochondrial function and intracellular localization. Wang et al. found that abnormal mitochondrial dynamics due to changes in mitochondrial fission/fusion proteins exist in AD brain [37]. In accordance with their findings, we found that mitochondrial fission-related protein (DRP1) was increased, while the fusion-related proteins (Mfn2 and OPA1) were decreased in the AD model. This phenomenon was significantly reversed by Ebselen treatment (Figure 6E,F).

The main function of mitochondria is to synthesize ATP. Aitken et al. found that cyanide or rotenone inactivate mitochondria, resulting in inhibition of neurotransmitter transmission in the presynaptic membrane [38]. Sudhof et al. proved that the release of neurotransmitters is an energy-dependent process. The loading, initiation and endocytosis of a neurotransmitter are all driven by ATP or GTP [39]. Zhu et al. found that adenosine release, H_2_O_2_ formation and ATP consumption inhibit synaptic activity in the case of mitochondrial inactivation [40]. In this study, we found that not only mitochondria but also synapses were damaged in the brains of AD models, which were all repaired by Ebselen to some extent. It is speculated that Ebselen might also enhance synaptic activity by repairing mitochondrial morphology and function, thereby improving synaptic function and interfering with AD.

The beneficial and harmful effect of selenium depends on its dose and form. The dosage range of Ebselen with positive effects is supposed to be very narrow. By analyzing all the measured indicators, we found in most cases that 0.5 and 1 μM Ebselen exhibited better improvement effects than 2 μM Ebselen did, with the only exception of mtTFA (Figure 6D) and DRP1 (Figure 6F). For example, the levels of Aβ plaques in the CA3 and cortex regions were much higher in 2 μM-Ebselen-treated mice than those of 0.5 or 1 μM groups (Figure 4C,D). Previously, Ebselen at 10 μM was found to significantly reduce the viability of hippocampal astrocytes [26]. In our study, although 2 μM Ebselen showed no toxic effect, as it did not change the levels of biochemical indicators related with damage of the liver, kidney, pancreas or glucose and lipid metabolism (Figure 1), its anti-AD therapeutic effect was indeed compromised.

## 5. Conclusions

In summary, we demonstrated that Ebselen rescued behavioral deficits of 3×Tg-AD mice, repaired synaptic damage and reduced production and deposition of Aβ. In addition, Ebselen alleviated the structural and functional damage of mitochondria in AD models by improving mitochondrial energy metabolism, balancing mitochondrial dynamics and enhancing mitochondrial biogenesis. Taken together, these results shed light on a novel mechanism of Ebselen in AD treatment. 

## Data Availability

The data presented in this study are available in the article or supplementary materials.

## Supplementary Materials

The following supporting information can be downloaded at: https://www.mdpi.com/article/10.3390/antiox11071350/s1. Figure S1. Ebselen (1.25, 2.5 and 5 μM) improved activity and expression levels of GPX in N2a-SW cells. Figure S2. (a) Total number of explorations in the Novel Object Recognition Test; (b) total number of arm entries in the Y Maze Test. Figure S3. Representative transmission electron microscopic images of mitochondria in N2a-SW cells. Figure S4. Ebselen improved mitochondrial energy metabolism, enhanced mitochondrial biogenesis and balanced mitochondrial fission/fusion in N2a-SW cells. Materials and Methods.
