# Supplementary material for: Ebselen Interferes with Alzheimer’s Disease by Regulating Mitochondrial Function
Source: Antioxidants (Basel). 2022 Jul 11;11(7):1350. doi: 10.3390/antiox11071350 (PMC9312019; doi:10.3390/antiox11071350)
Supplement: Supplementary file 1 [file antioxidants-11-01350-s001.zip › antioxidants-1749609-supplementary.pdf]

antioxidants

MDPI

Supplementary Materials: Ebselen Interferes with Alzheimer's Disease by Regulating Mitochondrial Function

Xuexia Li [1,2,3,4], Qingqing Shi [5], Hao Xu [1], Yufang Xiong [1], Chao Wang [6], Linfeng Le [1], Junliang Lian [1], Guoli Wu [1], Feiyuan Peng [1], Qiong Liu [1,2,3] and Xiubo Du [1,4,*]

1 Guangdong Provincial Key Laboratory for Plant Epigenetics, College of Life Sciences and Oceanography, Shenzhen University, Shenzhen 518055, China; xxli@szu.edu.cn (X.L.); 2019302013@email.szu.edu.cn (H.X.); 2019305009@email.szu.edu.cn (Y.X.); 2019301033@email.szu.edu.cn (L.L.); 2018304045@email.szu.edu.cn (J.L.); 2020302016@email.szu.edu.cn (G.W.); 2021300031@email.szu.edu.cn (F.P.); liuqiong@szu.edu.cn (Q.L.)

2 Key Laboratory of Optoelectronic Devices and Systems of Ministry of Education and Guangdong Province, College of Physics and Optoelectronic Engineering, Shenzhen University, Shenzhen 518060, China

3 Shenzhen-Hong Kong Institute of Brain Science-Shenzhen Fundamental Research Institutions, Shenzhen 518055, China

4 Shenzhen Bay Laboratory, Shenzhen 518055, China

5 Department of Psychiatry, Xijing Hospital, Air Force Medical University, Xi'an 710032, China; pengzw@fmmu.edu.cn

6 Shenzhen Center for Disease Control and Prevention, Shenzhen 518055, China; lihua@wjw.sz.gov.cn

* Correspondence: duxiubo@szu.edu.cn

This file includes:

1. Figures S1 to S4

2. Materials and Methods

1. Supplementary Figures

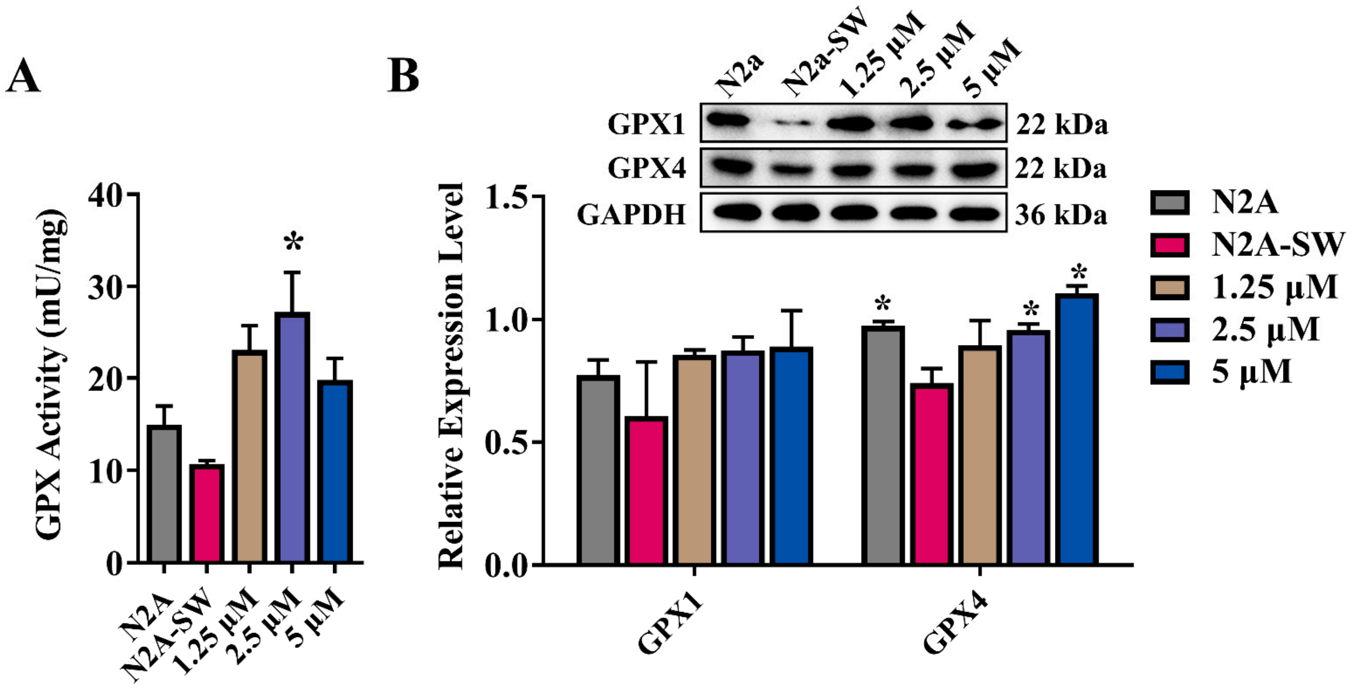

Figure S1. Ebselen (1.25 μM, 2.5 μM and 5 μM) improved activity and expression levels of GPX in N2a-SW cells: (**a**) Activities of GPX measured by Total Glutathione Peroxidase Assay Kit; (**b**) Representative western blot analysis of GPX 1 and GPX 4. The quantitative results were normalized against the levels of GAPDH. (* $p < 0.05$ vs. AD group; $n = 4$).

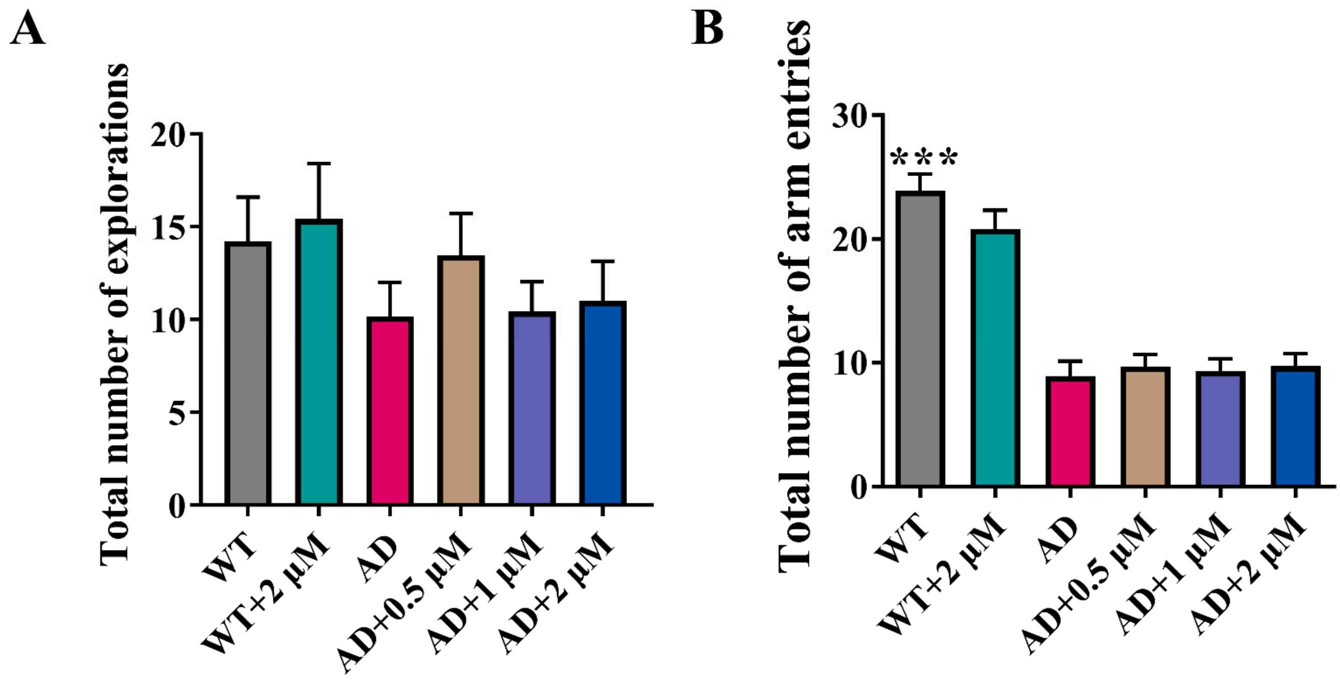

Figure S2. (**a**) Total number of explorations in Novel Object Recognition Test; (**b**) Total number of arm entries in Y Maze Test. (*** $p < 0.001$ vs. AD group; $n = 12$).

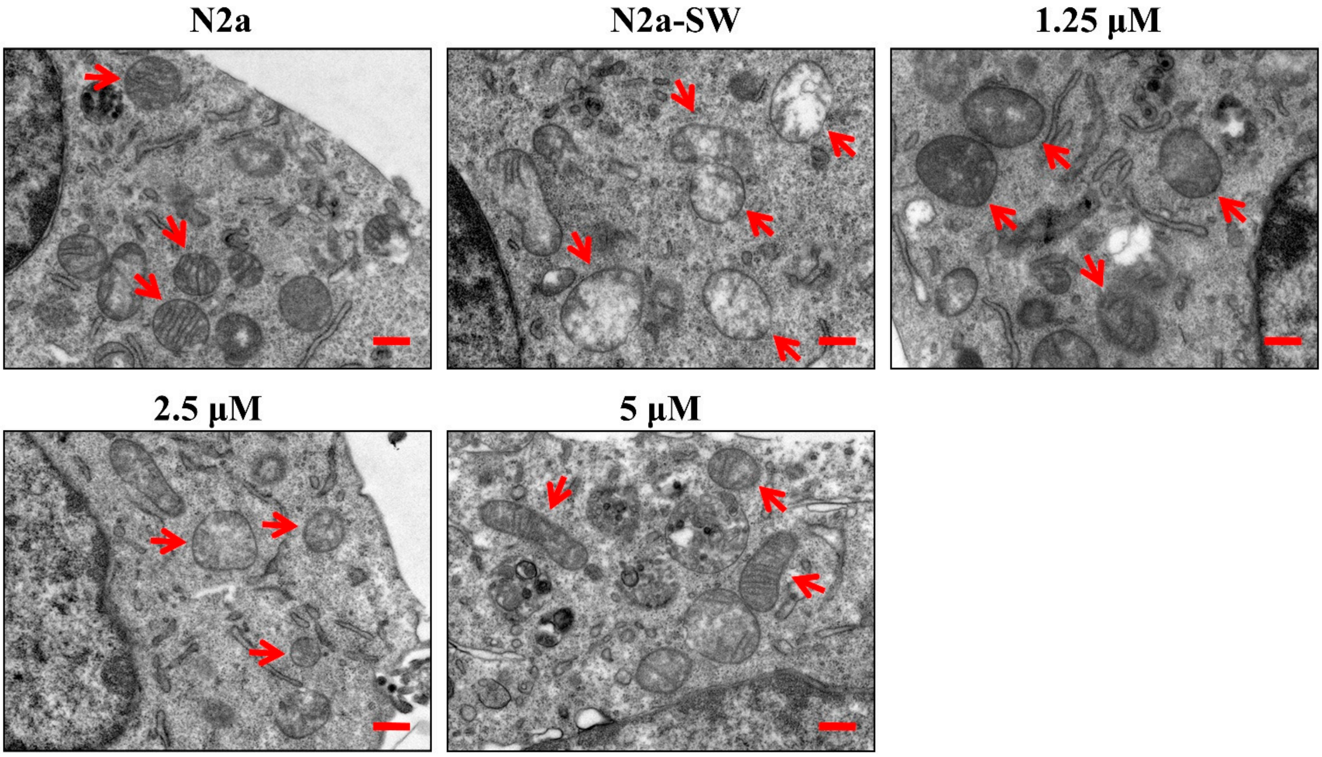

Figure S3. Representative transmission electron microscopic images of mitochondria (red arrows) in N2a-SW cells. (Scale bar: 5 μm; *n* = 3).

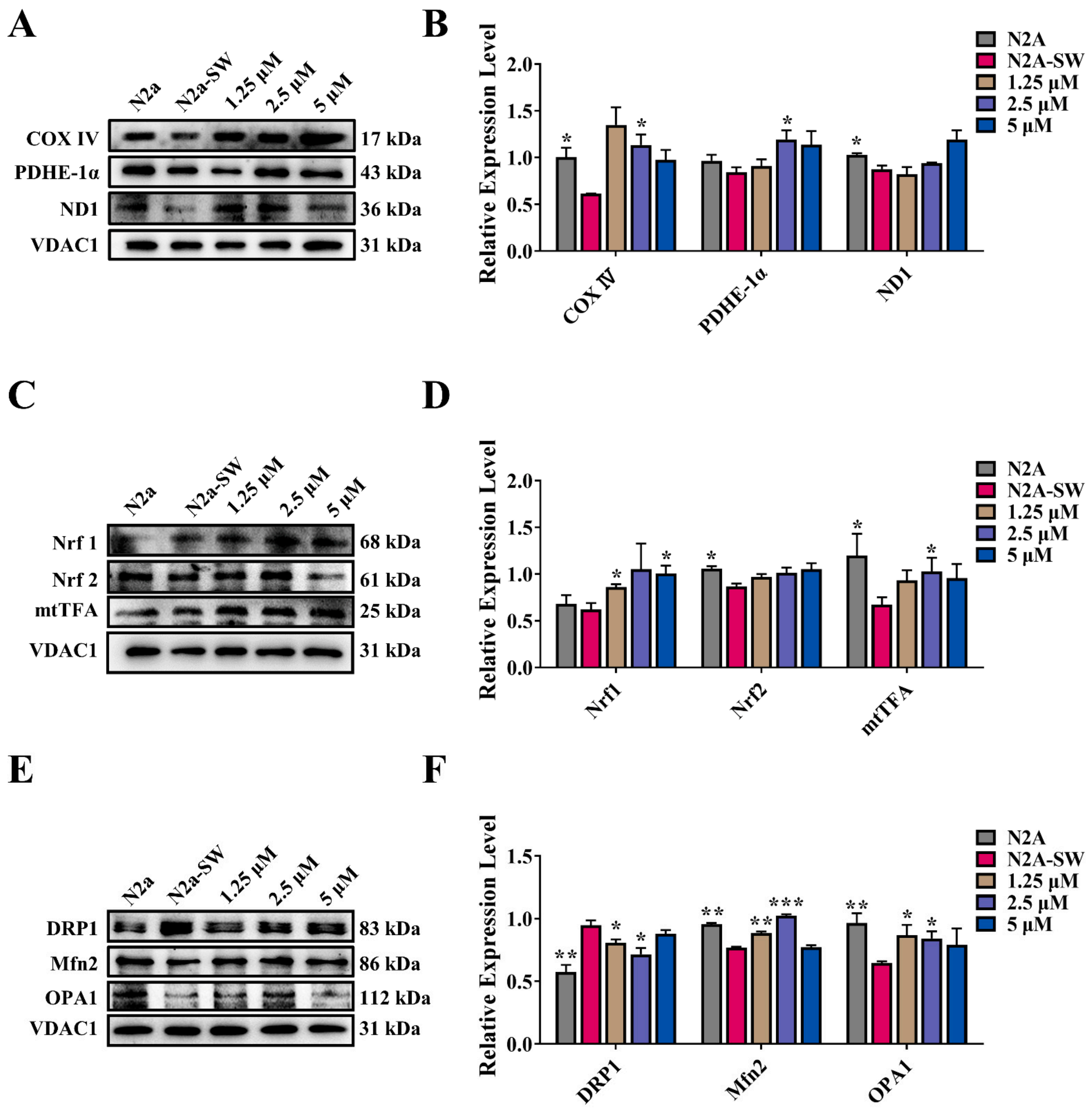

Figure S4. Ebselen improved mitochondrial energy metabolism, enhanced mitochondrial biogenesis, and balanced mitochondrial fission/fusion in N2a-SW cells: (**a**,**b**) Representative western blot analysis of mitochondrial energy metabolism-related proteins including COX IV, PDHE-1α and ND1; (**c**,**d**) Representative western blot analysis of mitochondrial biogenesis-related proteins including Nrf1, Nrf2, and mtTFA; (**e**,**f**) Representative western blot analysis of mitochondrial dynamics-related proteins including the fission protein Drp1, and the fusion proteins Mfn2 and OPA1. The quantitative results were normalized against the levels of VDAC1. (* $p < 0.05$, ** $p < 0.01$, *** $p < 0.001$ vs. AD group; $n = 4$).

2. Materials and Methods

2.1. Materials and Reagents

Wild-type murine neuroblastoma Neuro-2A (N2A) cells were got from Shanghai Institute of Biological Sciences, Chinese Academy of Sciences (Shanghai, China). Ebselen

and Thioflavin T (ThT) were bought from Sigma–Aldrich (Shanghai, China). Total Glutathione Peroxidase Assay Kit and Mitochondrial membrane potential assay kit with JC-1 were purchased from Beyotime Biotechnology (Shanghai, China). Antibodies of GPX 1, GPX 4, PSD95, COX IV, PDHE-1α, ND1, Drp1, Mfn2, OPA1, Nrf1, Nrf2, and mtTFA were acquired from Abcam (Shanghai, China). Biochemical reagents were got from Shenzhen Icubio Biomedical Technology Co., Ltd (Shenzhen, China). Primary antibody of 6E10 were purchased from Biolegend (Beijing, China). Seahorse XF Cell Mito Stress Test Kit was purchased from Agilent Technologies (Beijing, China).

2.2. Cell Culture and Treatments

N2A cells were cultured in a mixed medium containing 45% Opti-MEM, 49% dulbecco's modified eagle medium (DMEM), and 5% fetal bovine serum supplemented with 1% antibiotic (penicillin G and streptomycin) and incubated in an atmosphere with 5% CO_2 at 37 °C. N2a-APP695-swedish (N2a-SW) cells were N2a cells stably over-expressing with human Swedish mutant APP695, which were gifts from Prof. Zhang Yunwu (Xiamen University, China). N2a-SW cells were maintained in N2a growth medium supplemented with 0.2% G418. After treated with Ebselen dissolved in DMSO and diluted in serum free medium for 24 h, cells were collected for the following analysis.

2.3. Assay Kit-Related Operations

GPX activity, mitochondrial oxygen consumption, and mitochondria membrane potential ($\Delta\Psi m$) were detected strictly according to Total Glutathione Peroxidase Assay Kit, Mitochondrial membrane potential assay kit with JC-1, and Seahorse XF Cell Mito Stress Test Kit instructions, respectively.

2.3.1. Measurement of GPX Activity

After treated with Ebselen, cells were harvested and lysed. The samples were then homogenized followed by centrifugation at 12,000 rpm for 30 minutes at 4 °C. Supernatant was collected for total glutathione peroxidase (GPx) measurement. The protein content was quantified with the BCA protein assay kit.

2.3.2. Mitochondrial Oxygen Consumption Test

Briefly, 100 μl of cell suspension was plated into XF 24 polystyrene cell culture plates at the density of 10^5 cells per well and incubated for 4 h. Afterward, 150 μl of drug-containing medium was added to each well for another 24 h. On the day before the assay, a sensor cartridge was hydrated overnight in a non-CO_2 incubator. And assay medium was prepared by adding 2 mM glutamine, 10 mM glucose, and 1 mM pyruvate into Agilent Seahorse XF Base Medium, which was then maintained at 37 °C and adjust the pH to 7.4. The next day, cells were washed with the assay medium and incubated for 60 min at 37 °C in a non-CO_2 incubator before the assay started. At the same time, oligomycin (1 μM), FCCP (0.5 μM), and rotenone & antimycin A (0. 5 μM) was loaded in the respective ports and then calibrated in a Seahorse XFe24 Extracellular Flux Analyzer. Twenty minutes later, the cell culture plate was substituted with the sensor cartridge to get the basal oxygen consumption rate (OCR).

2.3.3. Measurement of $\Delta\Psi m$

After treatment with Ebselen for 24 h, cells were loaded with 1×JC-1 for 20 min at 37°C, washed twice with JC-1 buffer and then visualized using a confocal microscope.

2.4. Animals and Treatment

The final concentration of Ebselen in drinking water was 2 μM, 1 μM and 0.5 μM. 3×Tg-AD mice harboring TauP301L, PS1M146 V, and APPswe were purchased from Jackson laboratory (Bar Harbor, ME, USA). Age matched wild type (WT) mice were used as

controls. All mice were housed in 12h light/12h dark cycle at 22 ± 2 °C, with free access to water and food. 4-month-old mice were divided into six groups (n = 12 per group; six males and six females in each group): (1) vehicle-treated WT group; (2) 2 μM Ebselen-treated WT group; (3) vehicle-treated AD group; (4) 2 μM Ebselen-treated AD group; (5) 1 μM Ebselen -treated AD group; and (6) 0.5 μM Ebselen-treated AD group. Approximately 4.5 ml of water was consumed by each mouse per day. The average weight of each mouse was 25 g. Thus, 2 μM Ebselen-treated mice received a dose of approximately 98.72 ug/kg body weight or 0.36 umol/kg body weight per day.

These mice were administered with drinking water or Ebselen containing drinking water for 5 months, and then tested through a series of behavioral tests and sacrificed for further analysis. Animal experiments were approved by the Animal Ethical and Welfare Committee of Shenzhen University (Permit Number: AEWC-20140615-002).

2.5. Biochemical Analysis

Eyeballs blood was collected and centrifugated at 12,000 rpm for 20 min at 4 °C. Supernatant was collected and used for serum biochemical indexes measurement by a biochemical analyzer (iMagic-M7, China).

2.6. Behavioral Tasks

2.6.1. Morris Water Maze

The apparatus was a circular pool (120 cm in diameter and 40 cm in height) which was divided into four quadrants. A platform (10 cm in diameter) was submerged 1 cm below the surface of the water (20 ± 1 °C). Morris water maze test consisted of spatial learning trial and probe trials. The spatial learning trial included 5 consecutive trial days. The time that a mouse took to find the hidden platform was defined as escape latency. Upon failing to find the platform within 60 s, the mouse was manually guided to the platform and allowed to stay there for 10 s. Probe trials were performed 24 h and 72 h after the spatial learning trial, during which the platform was removed and mice were allowed to swim freely for 120 s. The time of the mice spent in the target quadrant, and the times mice crossed where the platform had been located were monitored.

2.6.2. Novel Object Recognition Test

On the first day, each mouse was habituated in the testing field (40 cm ×40 cm ×40 cm) for 5 mini. On the second day, two identical objects were placed equidistant from the center of the same field, and each mouse was allowed to move freely in the field for 5 min. On the third day, one of the objects was changed with a novel one. Each mouse was placed back in the field for 5 min. Objects and the field were cleaned with 75% ethanol between trials. The time spent exploring each object was monitored. New object exploration ratio was calculated as "(time spent exploring the novel object) / (time spent exploring both objects) ×100".

2.6.3. Y Maze Test

The apparatus of Y-maze consisted of three arms each measuring 30 cm long × 15 cm high × 8 cm wide. Each mouse was placed at the same place and allowed to freely explore all three arms for 5 minutes. The apparatus was cleaned with 75% ethanol between animals. Spontaneous alterations were defined as consecutively entered three different arms and calculated as "the number of spontaneous alterations/(the total number of arm entries − 2)." to avoid odor cues.

2.6.4. Elevated Plus Maze Test

The 'plus' sign-shaped device consisted of two opposite open arms (50 × 10 cm) and two opposite closed arms (50 × 10 × 15 cm) at a height of 40 cm. Each mouse was placed in the center facing the same arm and allowed to move freely for 5 min. Between trials,

the arena was cleaned with 75% ethanol. The number of open arms entries and the time spent in open arms were monitored.

2.6.5. Open Field Test

The apparatus is a plastic open field (40 cm × 40 cm × 40 cm). The floor is divided into 25 equal-sized grids. Each mouse was placed in the same place and moved freely for 3 min in the apparatus. Between trials, the arena was cleaned with 75% ethanol. The number of grids crossed with all paws, the number of rearing i.e., the animal stood on its hind legs, and the total distance were monitored.

2.7. Western Blot

After treated with Ebselen, cells or brain tissues were harvested and lysed. The samples were then homogenized and centrifugated at 12,000 rpm for 30 minutes at 4 °C. Supernatant was collected and the protein concentration was quantified by the BCA protein assay kit. Twenty micrograms of protein per lane were separated by SDS-PAGE and transferred to polyvinylidene fluoride membranes. Then the membrane was incubated with 5% fat-free milk for 1.5 h, primary antibodies overnight, and secondary antibody for 2 h. Between incubations, The membrane was washed 3 times with TBST. After sufficient washing, the bolts in the membrane were visualized by Image Station. Quantitative analysis was performed with Image J software.

2.8. Transmission Electron Microscope

The ultrastructure of synapses and mitochondria in cell lines and cortex of mice were observed by transmission electron microscopy (TEM). After fixed at room temperature for 2 h and then 4 °C overnight, samples were sent to Wuhan Sevier Biotechnology Co., Ltd for follow-up treatment, and viewed with TEM.

2.9. Thioflavin T Staining

Fresh tissues were fixed in 4% phosphate-buffered paraformaldehyde, embedded in OCT, serially sectioned (6 mm), and stored at –80 °C. Thioflavin T specifically binded to the layered structure of β-amyloid fibrils and emitted fluorescence after binding. Briefly, frozen sections were taken from the 5-min wash in PBS and placed in Thioflavin T solution (0.1 % in PBS) for 30 min. After washing with ultrapure water for 5 seconds, the sections were visualized by an upright fluorescence microscope (OLYMPUS BX51).

2.10. Statistical Analysis

All data were presented as mean ± standard error. Statistical analysis was analyzed through Student's t test, or one-way ANOVA followed by Bonferroni post-test through GraphPad Prism 7.0 software. The level of $p < 0.05$ (*), $p < 0.01$ (**), or $p < 0.001$ (***) were considered statistically significant.
